# Exopolysaccharide Produced by *Lactiplantibacillus plantarum*-12 Alleviates Intestinal Inflammation and Colon Cancer Symptoms by Modulating the Gut Microbiome and Metabolites of C57BL/6 Mice Treated by Azoxymethane/Dextran Sulfate Sodium Salt

**DOI:** 10.3390/foods10123060

**Published:** 2021-12-09

**Authors:** Fenglian Ma, Yinglong Song, Mengying Sun, Arong Wang, Shujuan Jiang, Guangqing Mu, Yanfeng Tuo

**Affiliations:** 1 School of Food Science and Technology, Dalian Polytechnic University, Dalian 116034, China; fenglianma@163.com (F.M.); sylong1016@163.com (Y.S.); smy493006403@163.com (M.S.); wangarong2021@163.com (A.W.); jiangsj@dlpu.edu.cn (S.J.); 2 Dalian Probiotics Function Research Key Laboratory, Dalian Polytechnic University, Dalian 116034, China

**Keywords:** exopolysaccharide, *Lactiplantibacillus plantarum*, gut microbiota, metabolite, colon cancer

## Abstract

Exopolysaccharide produced by *Lactiplantibacillus plantarum*-12 (LPEPS) exhibited the anti-proliferating effect on human colon cancer cell line HT-29 in vitro. The purpose of the study was to determine the alleviating effects of LPEPS on colon cancer development of the C57BL/6 mice treated by azoxymethane/dextran sulfate sodium salt (AOM/DSS). The C57BL/6 mice treated by AOM/DSS were orally administered LPEPS daily for 85 days. The results showed that LPEPS oral administration enhanced colon tight-junction protein expression and ameliorated colon shortening and tumor burden of the AOM/DSS treated mice. Furthermore, LPEPS oral administration significantly reduced pro-inflammatory factors TNF-α, IL-8, and IL-1β levels and increased anti-inflammatory factor IL-10 level in the serum of the AOM/DSS-treated mice. LPEPS oral administration reversed the alterations of gut flora in AOM/DSS-treated mice, as evidenced by the increasing of the abundance of *Bacteroidetes*, *Bacteroidetes*/*Firmicutes* ratio, *Muribaculaceae*, *Burkholderiaceae*, and *norank_o__Rhodospirillales* and the decreasing of the abundance of *Firmicutes*, *Desulfovibrionaceae*, *Erysipelotrichaceae*, and *Helicobacteraceae*. The fecal metabolites of the AOM/DSS-treated mice were altered by LPEPS oral administration, involving lipid metabolism and amino acid metabolism. Together, these results suggested that LPEPS oral administration alleviated AOM/DSS-induced colon cancer symptoms of the C57BL/6 mice by modulating gut microbiota and metabolites, enhancing intestine barrier, inhibiting NF-κB pathway, and activating caspase cascade.

## 1. Introduction

Colon cancer is the most common cancer of human beings. The incidence and mortality of patients with colon cancer is ranked third and second, respectively [1]. Colon cancer is seriously threatening human health and brings many adverse effects on human beings. Gut microbiota plays a pivotal role in human health. A number of previous studies have shown that intestinal flora and metabolites of patients with colon cancer are different from those of healthy individuals [2,3,4]. Colon cancer patients are accompanied by gut microbiota composition dysbiosis, including the decrease of the diversity of gut microbiota, the increase of the abundance of potentially harmful bacteria, and the decrease of the abundance of beneficial bacteria, which leads to gut metabolite changes and intestinal inflammation. Inflammatory bowel disease (IBD) is the most common inflammatory disease in the gastrointestinal tract caused by abnormal immunity, which mainly includes ulcerative colitis and Crohn’s disease [5]. Previous studies have demonstrated that IBD is an important cause of colon cancer [6].

It is reported that exopolysaccharide (EPS) produced by lactic acid bacteria (LAB) exhibited various physiological functions, including antioxidant, immune regulation, anti-tumor, anti-inflammation, and anti-viral, and lowering blood pressure [7]. EPS are not catabolized by the human digestive system but enter the cecum and colon, where the microbiota ferment EPS to produce beneficial substances, especially short-chain fatty acids (SCFA), also reducing pH, inhibiting pathogens growth, increasing the abundance of beneficial bacteria, providing energy for colonic epithelial cells, and enhancing intestinal barrier function [2,3,8,9,10]. A growing number of evidences have demonstrated that natural active substances could regulate intestinal flora, reduce inflammation, and alleviate colonic disease symptoms [11,12,13]. Previous studies reported that exopolysaccharides from microorganisms alleviated symptoms of colon cancer via modulating gut microbiota and metabolites, enhancing intestinal barrier function, inhibiting NF-κB signaling pathway, and reducing tumor burden [7,14,15,16,17].

In our previous studies, LPEPS was found to exert anti-proliferative effect on the human colon cancer cell line HT-29 in vitro [18]. In addition, LPEPS consisted of galactose, mannose, glucuronic acid, galactosamine, glucose, and xylose [19]. In this study, we will explore the effects of LPEPS on gut flora, fecal metabolites, colon cancer development, and inflammation in the AOM/DSS-treated C57BL/6 mice.

## 2. Materials and Methods

### 2.1. Material and Reagents

Radioimmunoprecipitation (RIPA) with phenylmethanesulfonyl fluoride (PMSF) buffer and bicinchoninic acid (BCA) protein kit were ordered from Solarbio Life Science, China. Trifluoroacetic acid (TCA) was attained from Shanghai Macklin Biochemical Co., Ltd., China. The following primary antibodies were used for the Western bot of colon tissue: phosphorylated p38 (p-p38) and phosphorylated NF-κB (p-p65) antibodies (Cell Signaling, Danvers, MA, USA), Inhibitor kappa B-α (IκB-α), NF-κB (p65), p38, Claudin-1, Caspase-8/9/3, Bax, proliferating cell nuclear antigen (PCNA), and β-actin antibodies (Beyotime Institute of Biotechnology, Shanghai, China).

### 2.2. Preparation of the LPEPS

LPEPS was isolated as previously described [18]. Briefly, *L. plantarum*-12 was cultured in MRS medium at 37 °C for 24 h. The cell-free supernatant was achieved by centrifugation (10,000× *g* for 10 min at 4 °C). To inactivate the enzyme, the cell-free supernatant was boiled for 10 min. The cell-free supernatant was concentrated to 1/4 of the original volume by rotary evaporator. In order to remove the protein, TCA was added to the supernatant to reach a final concentration of 4% (*w*/*v*) at room temperature with stirring for 3 h. After concentration (10,000× *g* for 10 min at 4 °C), LPEPS from the supernatant was precipitated by adding two times the volume of cold ethanol and was then stored at 4 °C for 24 h. LPEPS was obtained by concentration (10,000× *g* for 10 min at 4 °C). The LPEPS was re-suspended in deionized water and dialyzed against deionized water for 3 d (8000–14,000 Da, Yuan Ye Biological Technology, Shanghai, China), and the deionized water was changed every 8 h. The dialyzed retentate was lyophilized to obtain the crude LPEPS. Finally, the LPEPS was dissolved in normal saline at the concentration of 200 mg/kg body weight.

### 2.3. Animal Experiment Design

**Figure 1:**
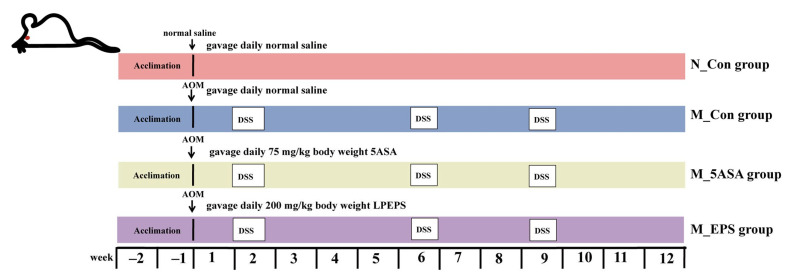
Experiment workflow of C57BL/6 colon cancer mice induced by AOM/DSS. N_Con group was intraperitoneally injected with normal saline, whereas the rest groups were intraperitoneally injected with 12.5 mg/kg body weight azoxymethane (AOM) at the start of the experiment (day 0). The mice in N_Con group were given fresh water every day, and the mice in other groups were given water containing 2.5% dextran sulfate sodium salt (DSS) for 5 days at the 2nd, 6th, and 9th weeks.

All animal experiments were carried out according to the Guidelines of Experimental Animal Ethics Committee of Dalian Polytechnic University (SYXK2017-0005). Sixty 6-week-old male C57BL/6 mice were ordered from Liaoning Changsheng Biotechnology Co., Ltd., China, and they were housed at 22 ± 2 °C, 50 ± 10% relative humidity in a 12 h light/dark animal room and fed ad libitum diet at the Animal Center of Dalian Polytechnic University, China. After 2 weeks of acclimation, the mice were randomly divided into four groups (*n* = 15, per group). As shown in Figure 1, normal control group (N_Con) was administered by gavage normal saline every day, model control group (M_Con) with gavage normal saline every day, model plus 5ASA group (M_5ASA) with gavage 75 mg/kg body weight 5ASA every day, and model plus LPEPS group (M_EPS) with gavage 200 mg/kg body weight LPEPS every day. The N_Con group was intraperitoneally injected with normal saline, and other groups were intraperitoneally injected with 12.5 mg/kg body weight azoxymethane (AOM, Sigma Chemical Co., St. Louis, MO, USA) at the start of the experiment (day 0). The mice in N_Con group were given fresh water every day, and the mice in other groups were given water containing 2.5% dextran sulfate sodium salt (DSS, MW: 40,000 Da, MP Biomedicals, Santa Ana, CA, USA) for 5 days at the 2nd, 6th, and 9th weeks. The body weight of mice was recorded once a week among experiment. After the experiment, blood samples of mice were obtained from mice eyeballs. After centrifugation (3000× *g*, 10 min, 4 °C), the mice serum samples were stored at −80 °C refrigerator. The mice were euthanized at 12 weeks, and the colon, spleen, thymus, and liver were obtained and weighed.

### 2.4. Histological and Biochemical Analysis

The colonic tissues of the C57BL/6 mice were quickly obtained and fixed at 4% paraformaldehyde for 2 days. After ethanol dehydration and paraffin embedding, the colon tissues were cut into 4-μm slices. The images were obtained by microscope (Olympus Optical Co., Ltd., Beijing, China) after xylene dewaxing and hematoxylin and eosin staining.

The serum inflammatory cytokines of the C57BL/6 mice, including IL-8, TNF-α, IL-1β, and IL-10, were measured by ELISA kits according to the operation manuals (Nanjing Jiancheng Bioengineering Institute, Nanjing, China).

### 2.5. Western Blot Analysis

The protein samples from C57BL/6 mice colon were extracted with lysate (RIPA: PMSF, 99:1, Solarbio Life Science, Beijing, China) according to the instruction. The protein concentration was determined by BCA protein kit (Solarbio Life Science, Beijing, China). An equal amount of protein was electrophoresed by 12% SDS-PAGE. Then, the protein was transferred to the PVDF membrane (Millipore, Darmstadt, Germany). The PVDF membrane was then blocked with Tris-buffered saline Tween 20 (TBST) containing 5% skim milk for 1 h at room temperature. Then, the membranes- were incubated with primary antibodies (1:1000) for 12 h at 4 °C. After washes with TBST, the membranes were incubated with horseradish peroxidase (HRP) conjugated secondary antibodies (1:1000) at room temperature for 1 h. The protein bands were detected by BeyoECL star Kit (Beyotime Institute of Biotechnology) according to instructions. The protein was visualized by chemiluminescence system with an image scanner (Azure C300, Azure Biosystems, Dublin, CA, USA). The expression of protein was quantified by NIH Image J software and normalized to β-actin.

### 2.6. Gut Microbiota Analysis

The 16S rRNA gene sequence analysis of the C57BL/6 mice fecal samples was performed as described previously [20]. Briefly, total bacterial DNA was extracted from 24 fecal samples of the C57BL/6 mice (six samples per group). The V3-V4 regions of the bacteria 16S rRNA gene were amplified via universal primer 338F (5′-ACTCCTACGGGAGGCAGCAG-3′) and 806R (5′-GGACTACHVGGGTWTCTAAT-3′). Equimolar concentrations of the purified amplicons were paired-end sequenced on an Illumina Miseq platform (Illumina, San Diego, CA, USA) according to the standard instructions of Majorbio Bio-Pharm Technology Co., Ltd. (Shanghai, China).

Raw fastq files were demultiplexed, quality-filtered by Trimmomatic, and merged by FLASH. Operational taxonomic units (OTUs) were clustered with 97% similarity cutoff using UPARSE (version 7.1, http://drive5.com/uparse/, accessed on 20 October 2021), and chimeric sequences were identified and removed using UCHIME. The taxonomic assignment was based on the Silva (SSU123) database. Rarefaction curve, coverage index, Shannon index, and Ace index of gut microbiota based on the OUT level were calculated using R software package. Principal component analysis (PCA) plot was produced with the adonis. Circle chart of the community abundance distribution at the phylum level and family level was analyzed using the R software package. Differential species compared between groups in the significant difference analysis using Wilcoxon rank-sum test. The linear discriminant analysis (LDA) effect size (LEfSe) was used to indicate the bacterial biomarkers from phylum to genus level, and LDA score >3.0.

### 2.7. Fecal Metabolomic Analysis

Metabolomics analysis of the C57BL/6 mice fecal samples was performed by LC-MS, as previously described [21], but slightly changed. In brief, 24 fecal samples of the C57BL/6 mice (six samples per group) were collected and stored at −80 °C. Fecal metabolites were extracted by 400 μL methanol/water (4:1) and 20 µL L-2-Chloro-phenylalanine (0.3 mg/mL) as the internal standard. Metabolites were checked by UPLC-Triple TOF system (AB SCIEX), equipped with a HSS T3 column (100 nm × 2.1 mm id, 1.8 µm; Waters, Milford, CT, USA). The quality control (QC) sample examined the repeatability of the analysis process (every 7 analysis samples). The raw data preprocessing was performed by progenesis QI (Waters Corporation, Milford, CT, USA) for baseline filtering, peak recognition, integration, retention time correction, and peak alignment, and finally, a data matrix of retention time, mass charge ratio, and peak intensity was obtained. Data matrix was used to remove the missing value with 80% rule, then to fill the gap value (minimum value to fill the gap in the original matrix). The response intensity of the essential spectral peak of the sample is normalized by the sum normalization method. The normalized data matrix was obtained. The variables with relative standard deviation (RSD > 30%) of QC samples were deleted. Additionally, logarithmic processing of log10 was carried out. Finally, the data matrix was obtained for subsequent analysis. A principal component analysis (PCA) was performed to visualize metabolic alterations among groups. Metabolites compared between groups in the significant difference analysis using Kruskal−Wallis H test. Pathway enrichment analysis was performed by Python software package scipy. stats. The results were performed on the Majorbio Cloud Platform (www.majorbio.com, accessed on 20 October 2021).

### 2.8. Statistical Analysis

Experiment data were analyzed by SPSS Statistics version 20.0 (IBM, Chicago, IL, USA) with Independent sample *t*-test and one-way ANOVA followed by Duncan’s post-hoc test. The data are presented as mean ± SD. When *p* < 0.05, the data were considered as significant differences.

## 3. Results

### 3.1. Effect of LPEPS Oral Administration on the Body Weight, Colon Length, and Tumor Numbers of the AOM/DSS-Treated C57BL/6 Mice

**Figure 2:**
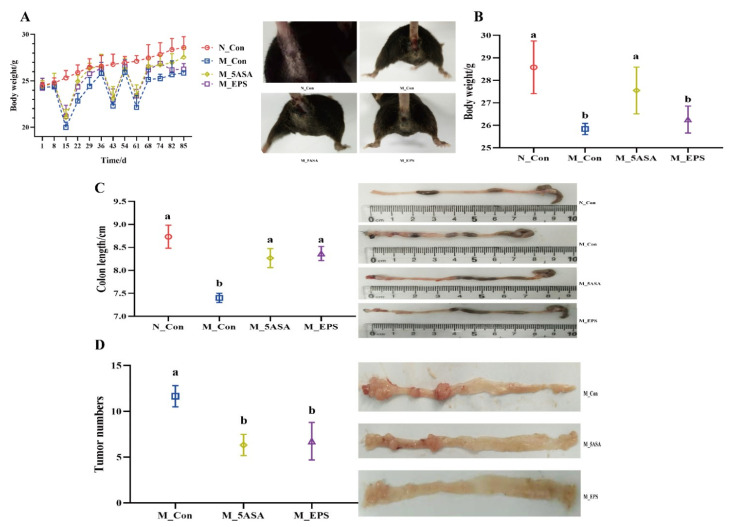
Effect of LPEPS oral administration on body weight, colon length, and tumor numbers of C57BL/6 colon cancer mice induced by AOM/DSS. (**A**) Body weight and morphology of mice, (**B**) body weight of mice on the eighty-fifth day, (**C**) colon length and colon morphology of mice, (**D**) tumor numbers and tumor morphology of mice. The data are presented as mean ± SD (*n* = 6). Different lowercase letters (a and b) are significantly different between groups (*p* < 0.05).

As shown in Figure 2A, the body weights of the C57BL/6 mice remarkedly decreased when they drank 2.5% DSS water in the second, sixth, and ninth weeks. On the eighty-fifth day, compared to the mice in M_Con group, the body weights of the mice in M_EPS group increased with no significant difference (Figure 2B). Colon length of the C57BL/6 mice in the M_Con group was significantly shorter than that of the mice in the N_Con group. However, LPEPS oral administration could restore the colon length of the C57BL/6 mice treated by AOM/DSS (Figure 2C). In addition, LPEPS oral administration significantly reduced tumor numbers in the AOM/DSS treated mice (Figure 2D).

### 3.2. Effect of LPEPS Oral Administration on Serum Inflammatory Cytokine and Organ Index of the AOM/DSS-Treated C57BL/6 Mice

**Figure 3:**
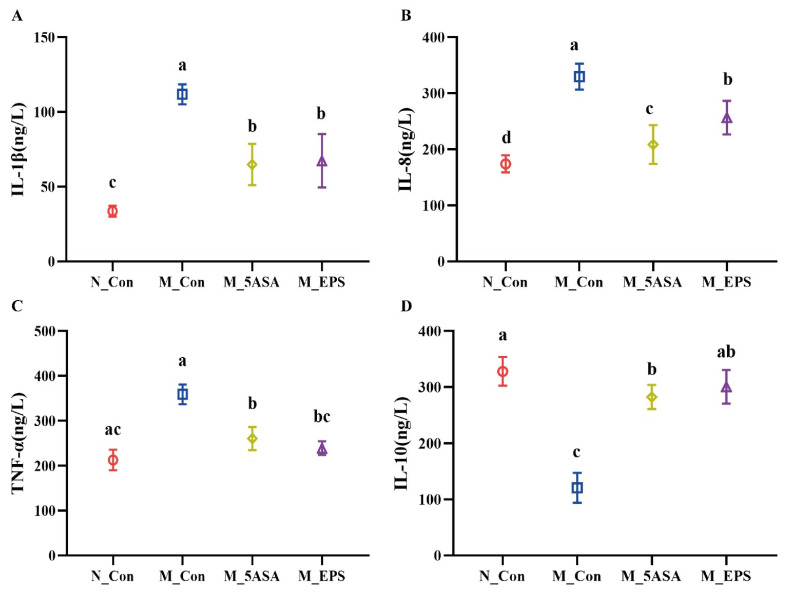
Effect of LPEPS oral administration on inflammatory factor of C57BL/6 colon cancer mice induced by AOM/DSS. (**A**) IL-1β, (**B**) IL-8, (**C**) TNF-α, (**D**) IL-10. The data are presented as mean ± SD (*n* = 4). Different lowercase letters (a, b and c) are significantly different between groups (*p* < 0.05).

**Figure 4:**
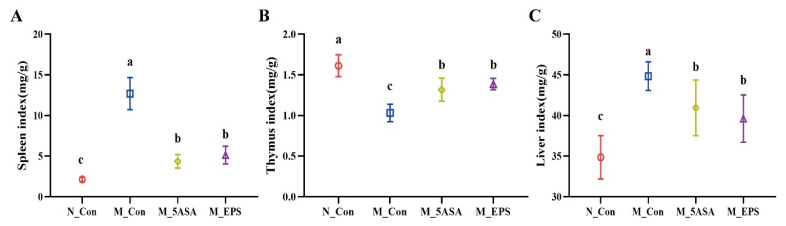
Effect of LPEPS oral administration on organ index of C57BL/6 colon cancer mice induced by AOM/DSS. (**A**) Spleen index, (**B**) thymus index, (**C**) liver index. The data are presented as mean ± SD (*n* = 6). Different lowercase letters (a, b and c) are significantly different between groups (*p* < 0.05).

As shown in Figure 3A–D, LPEPS oral administration regulated the expression of serum inflammatory factors of the AOM/DSS-treated C57BL/6 mice, as evidenced by down-regulating pro-inflammatory cytokines, IL-1β, IL-8, and TNF-α, and up-regulating anti-inflammatory cytokine IL-10 (*p* < 0.05). Furthermore, compared with N_Con group, the spleen index and liver index significantly increased, and thymus index decreased (*p* < 0.05) in the M_Con group, while LPEPS oral administration ameliorated the increase of spleen index and liver index and the decrease of thymus index (*p* < 0.05), as shown in Figure 4A–C.

### 3.3. Effect of LPEPS Oral Administration on Gut Barrier Function of the AOM/DSS-Treated C57BL/6 Mice

**Figure 5:**
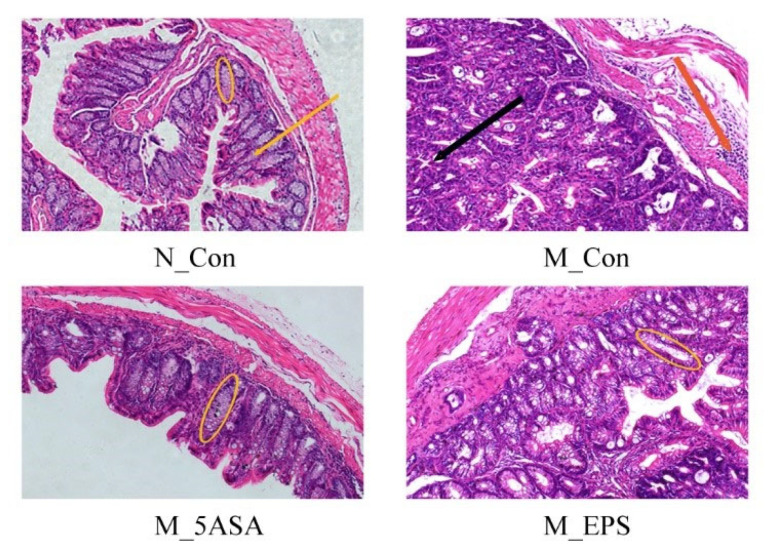
Effect of LPEPS oral administration on colonic histopathology of C57BL/6 colon cancer mice induced by AOM/DSS (*n* = 6). The yellow oval represents the colonic crypt. The yellow arrow represents goblet cells. Black arrow represents adenomas. The red arrow represents the inflammasome.

**Figure 6:**
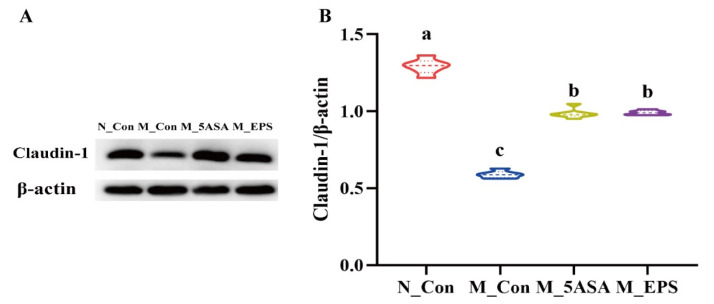
Effect of LPEPS oral administration on colonic tight-junction protein expression of C57BL/6 colon cancer mice induced by AOM/DSS. (**A**) The expression of colonic protein Claudin-1 was measured by western blot, with (**B**) β-actin as control for the protein blots (*n* = 4). Different lowercase letters (a, b and c) are significantly different between groups (*p* < 0.05).

As shown in Figure 5, compared with N_Con group, the number of goblet cells were decreased, and the crypt structure was changed in the AOM/DSS-treated mice colon tissue, while LPEPS oral administration could increase the number of goblet cells and restore the crypt structure. Furthermore, compared with the N_Con group, expression of the colonic tight-junction protein Claudin-1 was significantly reduced in the AOM/DSS-treated mice, while LPEPS oral administration alleviated the decrease of colonic tight-junction protein Claudin-1 (*p* < 0.05), as shown in Figure 6.

### 3.4. Effect of LPEPS Oral Administration on the Expression of Colonic Inflammation-Related Proteins of the AOM/DSS-Treated C57BL/6 Mice

**Figure 7:**
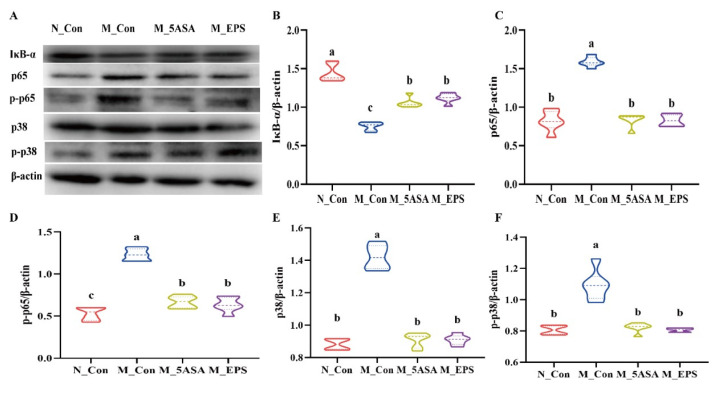
Effect of LPEPS oral administration on the expression of colonic inflammatory proteins in C57BL/6 colon cancer mice induced by AOM/DSS. (**A**–**F**) IκB-α, p65, p-p65, p38, and p-p38 in the colon of AOM/DSS-induced mice were determined by Western blot (*n* = 4). Different lowercase letters (a, b and c) are significantly different between groups (*p* < 0.05).

To explore the mechanism whereby LPEPS alleviated inflammation in the AOM/DSS-treated C57BL/6 mice, colonic inflammatory-related proteins were evaluated by Western blot. Compared with the N_Con group, the expression levels of p65, p-p65, p38, and p-p38 proteins were significantly increased, and the IκB-α level was decreased in the colon of the AOM/DSS-treated mice (*p* < 0.05), as shown Figure 7. However, LPEPS oral administration significantly alleviated the increase of p-p65 and p-p38 and the decrease of the IκB-α in the colon of the AOM/DSS-treated mice. As such, LPEPS oral administration alleviated colonic inflammation of AOM/DSS treated mice via inhibiting NF-κB and p38 MAPK signaling pathways.

### 3.5. Effect of LPEPS Oral Administration on the Expression of Colonic Apoptosis-Related Proteins of the AOM/DSS-Treated C57BL/6 Mice

**Figure 8:**
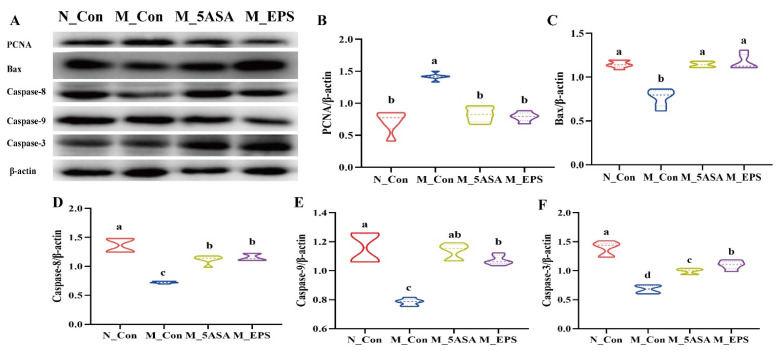
Effect of LPEPS oral administration on the expression of apoptosis-related proteins in C57BL/6 colon cancer induced by AOM/DSS. (**A**–**F**) PANA, Bax, caspase-8, caspase-9, and caspase-3 in the colon of AOM/DSS-induced mice were determined by Western blot (*n* = 4). Different lowercase letters (a, b and c) are significantly different between groups (*p* < 0.05).

To examine the mechanism of colon tumor apoptosis, the expression levels of colonic apoptosis-related proteins in the colon of the AOM/DSS-treated mice were evaluated by Western blot. Colonic protein PCNA level of AOM/DSS-treated mice was remarkedly increased, while LPEPS oral administration decreased the expression level of PCNA in the colon of the AOM/DSS-treated mice (Figure 8B). Furthermore, compared with N_Con group, the expression level of pro-apoptotic colonic protein Bax was significantly decreased in the M_Con group, while LPEPS oral administration increased Bax expression level (*p* < 0.05) in the AOM/DSS-treated mice, as shown in Figure 8C. In addition, LPEPS oral administration had significantly up-regulated expression of caspase-8, caspase-9, and caspase-3 proteins compared with M_Con group (Figure 8D–F). The results suggested that LPEPS oral administration promoted colon tumor apoptosis of the AOM/DSS-treated colon cancer mice through down-regulating PCNA and activating caspase cascade.

### 3.6. Effect of LPEPS Oral Administration on the Intestinal Flora of the AOM/DSS-Treated C57BL/6 Mice

**Figure 9:**
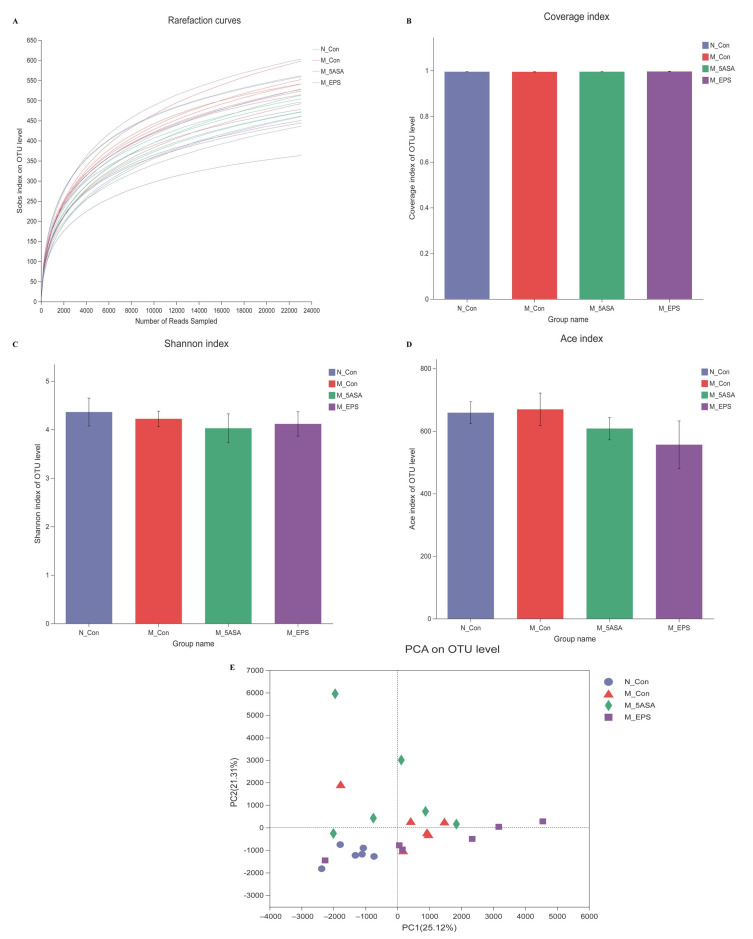
Effect of LPEPS oral administration on gut microbiota composition in C57BL/6 colon cancer mice induced by AOM/DSS (*n* = 6). (**A**) Rarefaction curves, (**B**) coverage index of OTU level, (**C**) Shannon index of OTU level, (**D**) Ace index of OTU level, (**E**) PCA analysis.

To explore the changes of intestinal microbiota structure and composition of C57BL/6 mice in each group, twenty-four fecal samples were examined by 16S rRNA gene sequencing approach. According to the results of the OTU level, rarefaction curve and coverage index indicate that depth of sequence was sufficient to explore the intestinal flora in four groups (Figure 9A,B). Through α diversity analysis, there was no significant difference in diversity and richness of intestinal microbiota (represented by the Shannon index and Ace index) between N_Con and M_Con groups (Figure 9C,D). Furthermore, principal component analysis (PCA) demonstrated that there was a difference between N_Con and M_Con groups at OTU level (Figure 9E). Moreover, M_EPS group could be separated from M_Con group, which indicated that LPEPS oral administration modulated the intestinal microbiota dysbiosis of AOM/DSS-treated mice.

**Figure 10:**
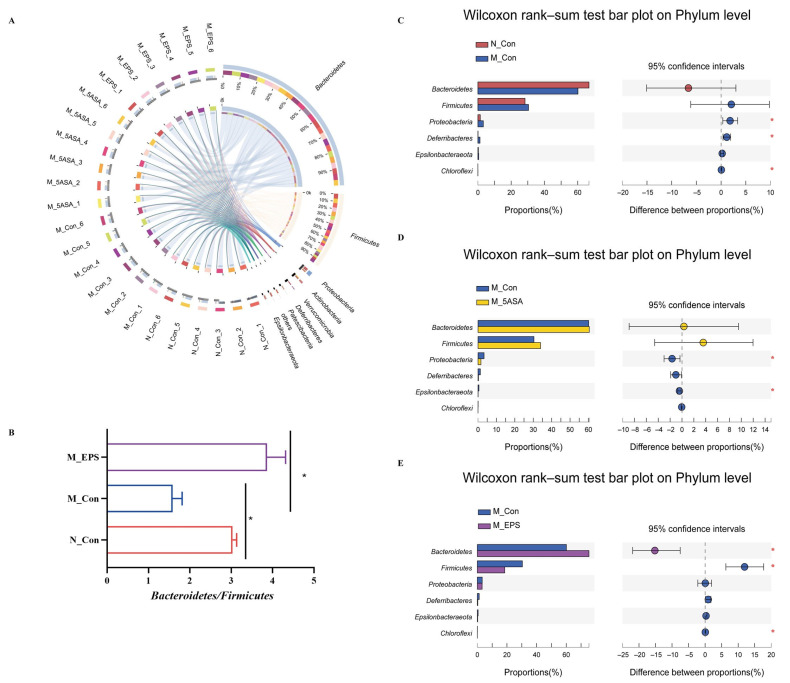
Effect of LPEPS oral administration on gut microbiota at the phylum level in C57BL/6 colon cancer mice induced by AOM/DSS (*n* = 6). (**A**) Circle picture, (**B**) *Bacteroidetes*/*Firmicutes* ratio, (**C**) N_Con vs. M_Con differential species analysis, (**D**) M_Con vs. M_5ASA differential species analysis, (**E**) M_Con vs. M_EPS differential species analysis. Red * *p* < 0.05 and black * *p* < 0.05.

**Figure 11:**
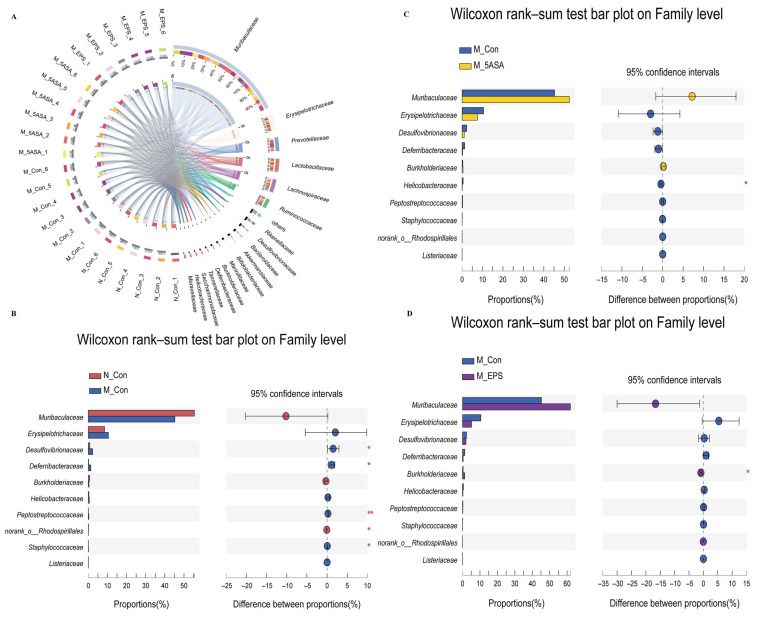
Effect of LPEPS oral administration on gut microbiota at the family level in C57BL/6 colon cancer mice induced by AOM/DSS (*n* = 6). (**A**) Circle picture, (**B**) N_Con vs. M_Con differential species analysis, (**C**) M_Con vs. M_5ASA differential species analysis, (**D**) M_Con vs. M_EPS differential species analysis. Red * *p* < 0.05, Red ** *p* < 0.01.

At the phylum level, *Bacteroidetes, Firmicutes*, *Proteobacteria, Actinobacteria, Verrucomicrobia*, and *Deferribacteres* were found to be the dominant fecal microbiota composition in the four groups of mice (Figure 10A). AOM/DSS treatment induced the increase in the abundance of *Proteobacteria*, *Deferribacteres*, *Chloroflexi*, *Firmicutes*, and *Epsionbacteraeota* and the decrease in *Bacteroidetes* and *Bacteroidetes*/*Firmicutes* ratio as compared with those in N_Con group mice (Figure 10B,C). Fortunately, LPEPS oral administration reversed the trend at the phylum level in the AOM/DSS-treated mice (Figure 10E). At the family level, the relative abundance of *Muribaculaceae*, *Burkholderiaceae*, and *norank_o__Rhodospirillales* was decreased, whereas the relative abundance of *Desulfovibrionaceae*, *Erysipelotrichaceae*, *Deferribacteraceae*, *Peptostreptococcaceae*, *Staphylococcaceae, Helicobacteraceae*, and *Listeriaceae* was increased in the M_Con group compared with that in the N_Con group, as shown in Figure 11B. Interestingly, the relative abundance of *Muribaculaceae*, *Burkholderiaceae*, and *norank_o__Rhodospirillales* was increased, and that of *Erysipelotrichaceae, Desulfovibrionaceae*, *Helicobacteraceae*, *Peptostreptococcaceae*, *Staphylococcaceae*, and *Listeriaceae* was decreased in the M_EPS and M_5ASA groups compared with M_Con group, as shown in Figure 11D.

**Figure 12:**
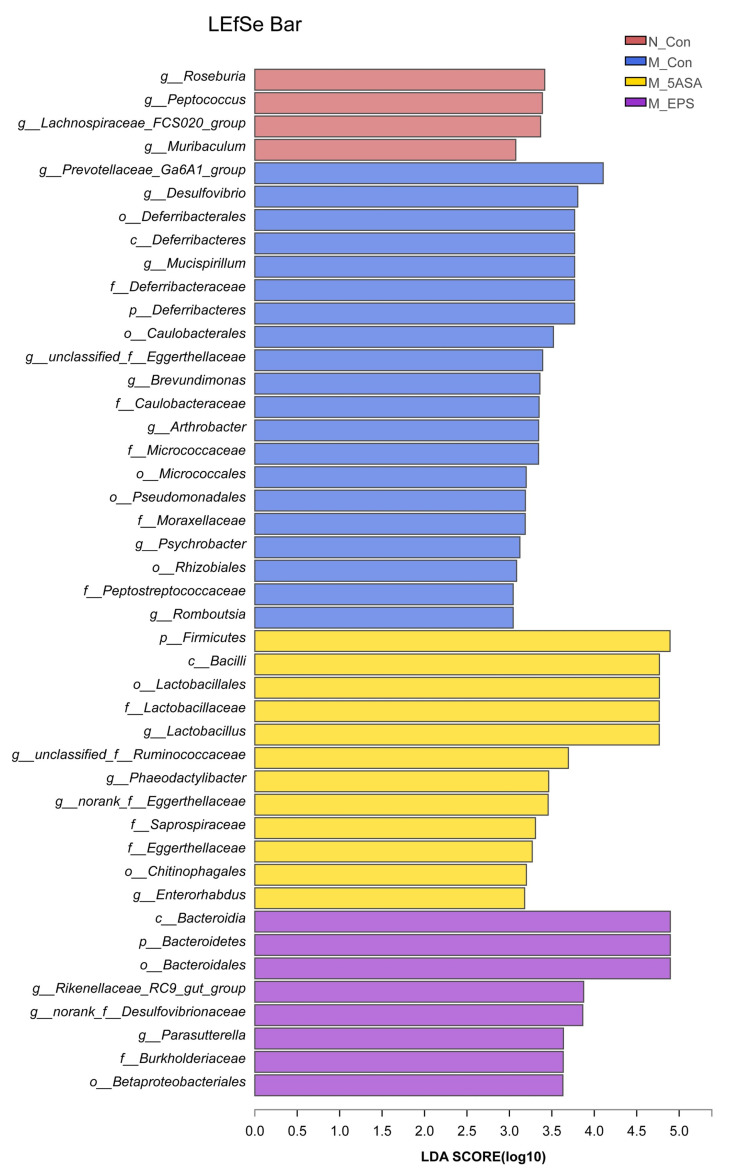
LEfSe analysis of gut microbiota in C57BL/6 colon cancer mice induced by AOM/DSS (*n* = 6). Biomarker taxa generated from LEfSe analysis (LDA > 3).

In addition, we analyzed the dominant microbiota in each group using linear discriminant analysis effect size (LEfSe) as shown in Figure 12. The results indicated that *Roseburia*, *Peptococcus*, and *Lachnospiraceae_FCS020_group* were dominant in the N_Con group. *Prevotellaceae_Ga6A1_group*, *Desulfovibrio*, and *Deferribacterales* were enriched in the M_Con group, while *Bacteroidia* and *Bacteroidetes* were the dominant in the M_EPS group.

### 3.7. Effect of LPEPS Oral Administration on the Fecal Metabolites of the AOM/DSS-Treated C57BL/6 Mice

**Figure 13:**
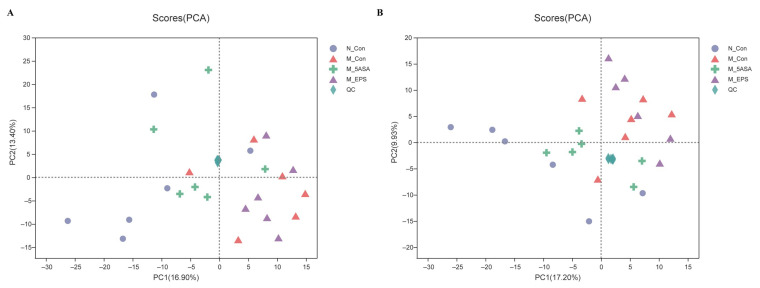
Untargeted fecal metabolomic analysis of C57BL/6 colon cancer mice induced by AOM/DSS (*n* = 6). (**A**) PCA score plot under negative ion mode. (**B**) PCA score plot under positive ion mode.

**Figure 14:**
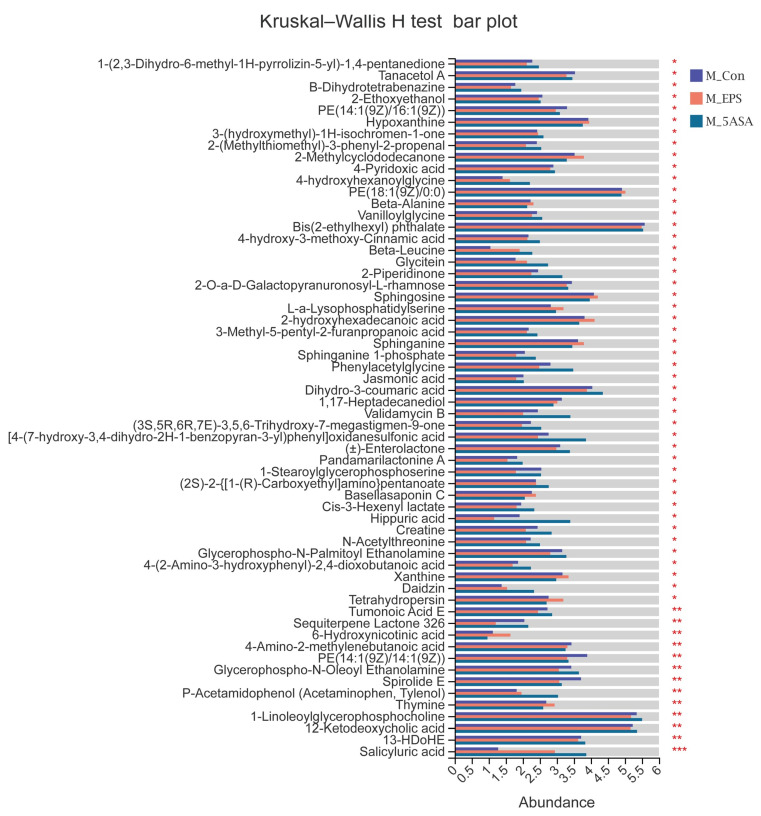
Differential metabolites analysis of C57BL/6 colon cancer mice induced by AOM/DSS in M_Con, M_5ASA, and M_EPS groups (*n* = 6). * 0.01 < *p* ≤ 0.05, ** 0.001 < *p* ≤ 0.01, *** *p* ≤ 0.001.

**Figure 15:**
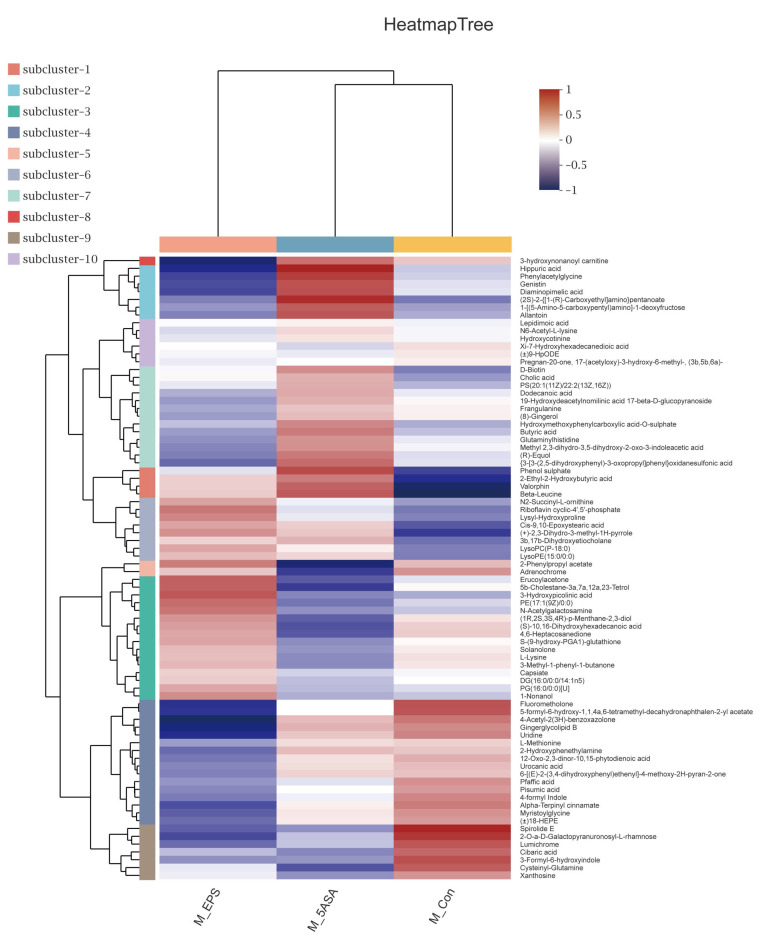
Cluster analysis metabolites of C57BL/6 colon cancer mice induced by AOM/DSS in M_Con, M_5ASA, and M_EPS groups (*n* = 6).

**Figure 16:**
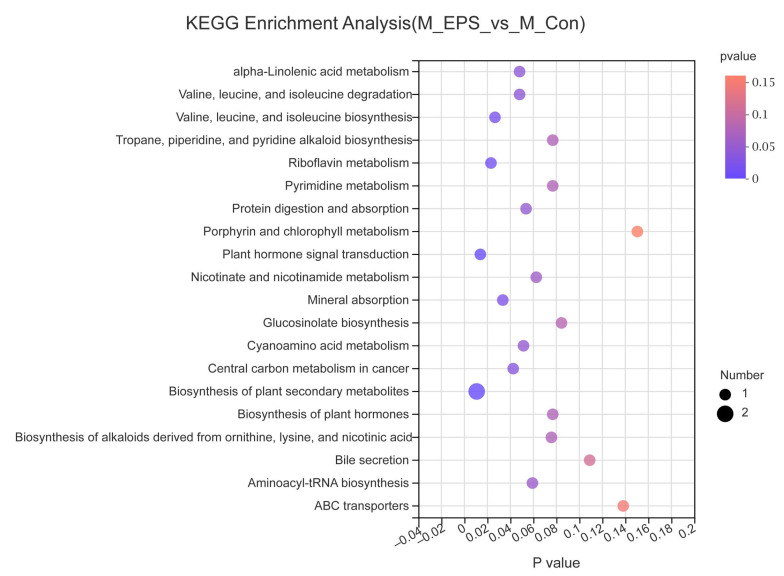
Metabolic pathway enrichment analysis of identified different metabolites of C57BL/6 colon cancer mice induced by AOM/DSS between M_Con and M_EPS group (*n* = 6). Generally, *p*-value less than 0.05 is considered as a significant enrichment term. The size of the bubble in the figure represents the number of metabolites enriched into the pathway.

To determine the role of LPEPS oral administration on gut metabolic profiles in AOM/DSS treated mice, PCA analysis was performed using data from N_Con, M_Con, M_5ASA, and M_EPS groups. PCA score plots showed the obvious separation trend between N_Con and M_Con group in both negative and positive ion modes (Figure 13A,B), and there was no significant separation between M_EPS group and M_Con group in both negative and positive ion modes (Figure 13A,B). The lysyl-hydroxyproline, 4-hydroxyhexanoylglycine, beta-alanine, beta-leucine, glycitein, and daidzin were obviously enriched by LPEPS oral administration; furthermore, the PE(14:1(9Z)/16:1(9Z)), 1-Stearoylglycerophosphoserine, PE(14:1(9Z)/14:1(9Z)), 1-Linoleoylglycerophosphocholine, xanthosine, uridine, 4-formyl indole, 3-Formyl-6-hydroxyindole, and 3-hydroxynonanoyl carnitine were evidently reduced by LPEPS oral administration compared to M_Con group (Figure 14 and Figure 15). The metabolite pathways analysis suggested that these metabolite pathways were mainly involved in valine, leucine, and isoleucine degradation; valine, leucine, and isoleucine biosynthesis; alpha-linolenic acid metabolism; central carbon metabolism in cancer; and mineral absorption (Figure 16).

### 3.8. Correlation Analysis

**Figure 17:**
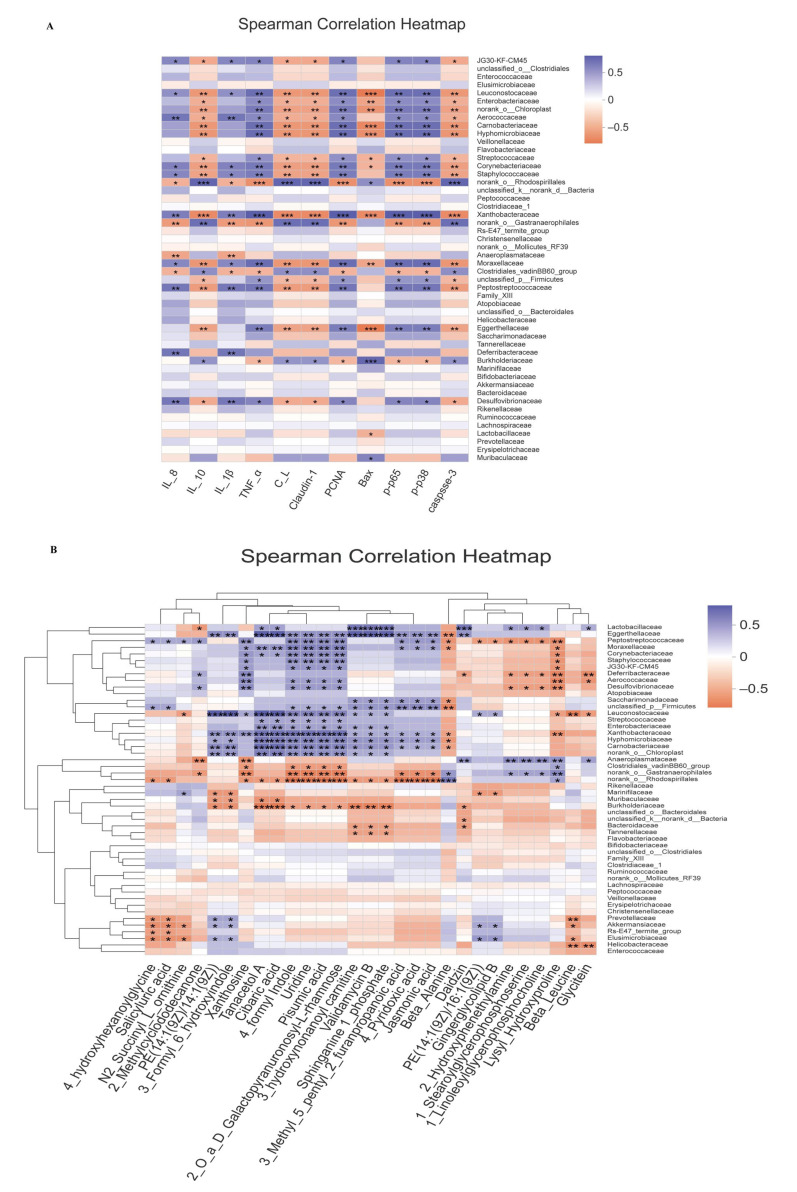
Correlation analysis. (**A**)The top 50 at the family level were correlated with the host phenotypes (*n* = 4). (**B**) The top 50 at the family level were correlated with the metabolites (*n* = 4). C_L, colon length; T_N, tumor numbers; B_W, body weight. Blue and Orange-red represent positive and negative correlations, respectively. * 0.01 < *p* ≤ 0.05, ** 0.001 < *p* ≤ 0.01, *** *p* ≤ 0.001.

The correlation between gut microbiota and host phenotypes was analyzed using Spearman’s correlation coefficient (Figure 17A). At the family level, the increased abundance of *Desulfovibrionaceae*, *Deferribacteraceae*, *Peptostreptococcaceae*, and *Staphylococcaceae* had significant positive correlation with the expression of intestinal pro-inflammatory cytokines IL-8, IL-1β, and tumor numbers, whereas increased level of IL-10, colon length, Claudin-1, and caspase-3 had significant negative relationship with *Desulfovibrionaceae*, *Peptostreptococcaceae*, *Xanthobacteraceae*, *Staphylococcaceae*, and *Enterobacteriaceae*. Meanwhile, the increased expression level of Bax was positively correlated with *Muribaculaceae*. The increased pro-inflammatory protein expression levels of PCNA, p-p65, and p-p38 were inversely associated with *Burkholderiaceae* and *norank_o__Rhodospirillales* and positively associated with *Desulfovibrionaceae* and *Enterobacteriaceae*.

Furthermore, we analyzed the correlation between fecal metabolites and gut microbiota in AOM/DSS-treated mice (Figure 17B). *Helicobacteraceae* was negatively correlated with beta_leucine and glycitein. The increased abundance of *Desulfovibrionaceae* was positively correlated with 4_formyl indole, uridine, pisumic acid, 2_O_a_D_Galactopyranuronosyl-L-rhamnose, and xanthosine but negatively correlated with lysyl_hydroxyproline. In addition, the abundance of *Muribaculaceae* and *Burkholderiaceae* were negatively correlated with tanacetol A, cibaric acid, PE(14:1(9Z)/14:1(9Z)), and 3_Formyl_6_hydroxyindole.

## 4. Discussion

In the present study, we used AOM/DSS-treated C57BL/6 mice to examine the ameliorating effect of LPEPS oral administration on the intestinal inflammatory disease and colon cancer development of the C57BL/6 mice. The results showed that LPEPS oral administration could modulate the intestinal flora and fecal metabolites, enhance intestinal barrier, and alleviate colon inflammation and colon tumor in AOM/DSS-treated C57BL/6 mice.

Intestinal flora plays a vital role in human health [22]. A growing number of studies have confirmed the gut microbiota imbalance and potential pathogenic bacteria increment of colon cancer patients [2,23,24]. In the present study, the results showed that an altered gut microbiome composition in AOM/DSS-treated mice characterized by imbalanced *Bacteroides*/*Firmicutes* ratio. However, compared with M_Con group, LPEPS oral administration remarkedly increased the *Bacteroides*/*Firmicutes* ratio, indicating that LPEPS oral administration restored intestinal flora. Ji et al. reported that jujube polysaccharides ameliorated AOM/DSS-induced gut microbiota dysbiosis by increasing ratio of *Bacteroidetes*/*Firmicutes* [8]. Li et al. also found that probiotics and prebiotics could quickly increase the *Bacteroides*/*Firmicutes* ratio, inhibit harmful bacteria growth, and accelerate the recovery of beneficial gut microbiota [25].

The relative abundance of *Bacteroidetes*, *Muribaculaceae*, *Burkholderiaceae*, and *norank_o__Rhodospirillales* in the intestine of AOM/DSS-induced colon cancer mice increased after treatment by LPEPS, while the relative abundance of *Firmicutes*, *Desulfovibrionaceae*, *Erysipelotrichaceae*, *Deferribacteraceae*, *Peptostreptococcaceae*, *Staphylococcaceae*, *Helicobacteraceae*, and *Listeriaceae* was decreased. *Muribaculaceae*, belonging to the phylum *Bacteroidetes*, is the dominant family in the intestine of mice, which has a wide range of uses in the degradation of complex carbohydrates [26]. Previous studies showed that the abundance of *Muribaculaceae* was significantly correlated with propionate [9,10,27]. This latter is well known to improve intestinal epithelial health [3]. *Desulfovibrionaceae* is one sulfate-reducing bacteria and produces metabolites H_2_S [28]. A number of studies showed that *Desulfovibrionaceae* could induce inflammation, injury gut mucosa, and decrease of colon barrier function [20,29,30]. Similarly, Luo et al. reported that the relative abundance of *Desulfovibrionaceae* was found to increase in the AOM/DSS-induced colon cancer mice and decrease due to the treatment of Ganoderma lucidum polysaccharide [13]. *Erysipelotrichaceae* played an important role in diseases associated with gastrointestinal inflammation and was significantly enriched in colon cancer hosts [31,32,33,34]. Previous studies have shown that *Erysipelotrichaceae* could promote inflammation in the intestine of colon cancer patients [4,35]. *Peptostreptococcaceae* was significantly enriched in gastrointestinal disease hosts; interestingly, naturally active substances could remarkedly reduce its abundance and down-regulate inflammation [36,37,38,39,40]. Studies have revealed that *Staphylococcaceae* was consistently augmented in IBD and colon cancer [2,23]. Zhou et al. reported that exopolysaccharides produced by *Lactiplantibacillus plantarum* NCU116 significantly decreased the relative abundance of *Staphylococcaceae* in the fecal of DSS-induced C57BL/6 mice [41]. Our results suggested that oral LPEPS were beneficial to maintain the gut microbiota balance and promote intestinal health of the C57BL/6 mice treated by AOM/DSS.

The microbiome dysbiosis leads to the disruption of fecal metabolome [3,4]. LPEPS oral administration decreased indoles contents, including 4-formyl indole and 3-Formyl-6-hydroxyindole, in the feces of AOM/DSS-treated C57BL/6 mice. Correlation analysis found that 4-formyl indole was positively correlated with *Desulfovibrionaceae*. 3_Formyl_6_hydroxyindole was negatively correlated with *Muribaculaceae*. Indole is the precursor of indoxyl sulfate, which linked with cancer, host metabolic disorders, cardiovascular disease, type 2 diabetes, hypertension, and induced intestinal barrier dysfunction [42,43,44,45,46,47]. It was reported that dietary supplements of beneficial substances effectively decreased indole-derived metabolites in the host [48,49,50]. LPEPS oral administration remarkedly decreased purine and pyrimidine nucleosides, including xanthosine and uridine, in the feces of AOM/DSS-treated C57BL/6 mice. In addition, uridine and xanthosine were negatively correlated with *Desulfovibrionaceae*. A growing number of studies have shown that uridine and xanthosine were significantly higher in colorectal cancer patients than in healthy humans [51,52,53,54]. LPEPS oral administration decreased 3-hydroxynonanoyl carnitine in the AOM/DSS-treated mice. A number of studies have shown that carnitine was enriched in colon cancer and type 2 diabetes [46,55,56,57,58], while natural active substance supplementation effectively decreased concentration of carnitine in type 2 diabetes [46,50].

LPEPS oral administration decreased glycerophospholipids, including PE(14:1(9Z)/16:1(9Z)), 1-Stearoylglycerophosphoserine, PE(14:1(9Z)/14:1(9Z)), and 1-Linoleoylglycerophosphocholine. PE(14:1(9Z)/14:1(9Z)) was negatively correlated with *Muribaculaceae* and *Burkholderiaceae*. Glycerophospholipids are the main lipid constituents of cell membranes and play a vital role in cell proliferation, cell differentiation, and cell apoptosis [59]. Emerging evidences implicated that glycerophospholipids were associated with insulin resistance, Type 2 diabetes and chronic kidney disease [60,61]. LPEPS oral administration increased isoflavonoids, including glycitein and daidzin. Glycitein was negatively correlated with *Helicobacteraceae*. To our knowledge, isoflavonoids have a great deal of versatile health-promoting benefits, such as anti-inflammatory, antioxidant, and anticancer properties [62,63,64]. LPEPS oral administration increased lysyl-hydroxyproline, beta-alanine and beta-leucine. Beta_leucine was negatively correlated with *Helicobacteraceae*. Lysyl_hydroxyproline was negatively correlated with *Desulfovibrionaceae*. Hydroxyproline could scavenge oxidants and regulate cell redox [65,66]. It was reported that alanine and leucine decreased in colorectal cancer [67,68]. Wang et al. reported that probiotics combined with prebiotic supplementation upregulated alanine and regulated amino acid metabolism in weaned rats [69].

Changes in community structure of the gut microbiome and fecal metabolites could affect disease severity in the host [3,43,70]. AOM/DSS-treated mice were associated with the loss of intestinal barrier integrity, changes immune organs, and increased inflammatory markers [71,72]. Claudin-1 is mainly responsible for intestinal barrier function, which maintains the integrity and permeability of intestine [73]. LPEPS oral administration promoted gut barrier function by increasing the expression of colonic tight-junction protein Claudin-1. Similarly, exopolysaccharides from *L. plantarum* NCU116 supplementation enhanced gut barrier function and tight-junction proteins expression in DSS-induced colitis mice [74]. LPEPS oral administration alleviated spleen hypertrophy and thymus atrophy symptoms in AOM/DSS-treated colon cancer mice. Spleen enlargement and thymus atrophy were common symptoms in mice with DSS-treated colitis and AOM/DSS-induced colon cancer [71,72,75,76], while tea polysaccharides supplementation could relieve the symptoms of splenomegaly and thymus atrophy in the AOM/DSS-treated mice [72]. Spleen and thymus are well known to be vital immune organs that can respond to the degree of inflammation [75,77,78]. LPEPS oral administration significantly down-regulated pro-inflammatory factors (IL-8, TNF-α, and IL-1β) and up-regulated anti-inflammatory factor (IL-10) levels in the serum of AOM/DSS-induced colon cancer mice. In addition, IL-8 and IL-1β had significant positive correlation with *Desulfovibrionaceae*, *Deferribacteraceae*, *Peptostreptococcaceae*, and *Staphylococcaceae*. Similarly, Song et al. reported that polysaccharides from *Rhizopus nigricans* could reduce pro-inflammatory factors in the AOM/DSS-treated mice [79].

AOM/DSS-induced mice were characterized by shortening colon length, increasing tumor burden, and loss of body weight [71,80]. A growing number of evidences suggested natural active substances alleviated tumor burden, colon length, and inflammation. It is widely known that NF-κB and p38MAPK play major role in the occurrence and development of cancer [81,82,83]. The NF-κB signaling pathway is activated by extracellular signaling factors, such as inflammatory factors, chemokines, and so on [84]. The activation of NF-κB increased pro-inflammatory factors, such as IL-6, IL-8, and TNF-α, further promote the inflammatory response [85]. In the present study, NF-κB was activated due to AOM/DSS-treatment, and this activated NF-κB induced the transcription of pro-inflammation factors, such as IL-β, IL-8, and TNF-α. LPEPS oral administration significantly inhibited NF-κB signaling pathway activity by down-regulating p-p65 protein expression, further reducing inflammatory response. Similarly, Bagheri et al. reported that *Brucea javanica* fruit extract induced HT-29 cells apoptosis by suppression of NF-κB pathway [86]. LPEPS oral administration significantly reduced expression of anti-apoptotic protein PCNA and increased expression of pro-apoptotic protein Bax. In addition, correlation analysis found that Bax was positively correlated with *Muribaculaceae*. PCNA was inversely associated with *Desulfovibrionaceae*. Similarly, Song et al. reported that an extracellular polysaccharide of *Rhizopus nigricans* notably enhanced Bax and reduced PCNA expression in AOM/DSS-induced mice [79]. Furthermore, LPEPS oral administration induced tumor apoptosis by activating caspase cascade, including caspase-8, caspase-9, and caspase-3. It has been reported that caspase activation is one of the major mechanisms of colon cancer cells apoptotic process. Similarly, Chen et al. reported that exopolysaccharide of Antarctic bacterium *Pseudoaltermonas* sp.S-5 induced apoptosis in the K562 cells via up-regulating the activities of caspase-9 and caspase-3 [87]. Taken together, LPEPS oral administration promotes the tumor apoptosis by down-regulating PCNA and up-regulating caspase cascade.

## 5. Conclusions

In this study, LPEPS oral administration for 85 days was found to alleviate the colon cancer symptoms of C57BL/6 mice treated by AOM/DSS. The results showed that LPEPS manipulated the gut microbiota and metabolites, enhanced gut barrier function, and alleviated colon cancer symptoms of AOM/DSS-treated mice by inhibiting inflammatory signaling and activating caspase cascade. Taken together, LPEPS could be used as a potential active substance to relieve inflammation and colon cancer burden of the colon cancer patients. However, the use of a small number of animals is a limitation of the study. In the future research, we will continue to expand the number of mice for follow-up experiments. Furthermore, the relationship between structure characteristics and function of LPEPS will be elucidated for the potential application as functional food additive.

## Data Availability

The data presented in this study are available on request from the corresponding author.
